# Synthesis, characterization and *in vitro* biological evaluation of two matrine derivatives

**DOI:** 10.1038/s41598-018-33908-8

**Published:** 2018-10-24

**Authors:** Xingan Cheng, Jingmin Ye, Huiqing He, Zhanmei Liu, Chunbao (Charles) Xu, Bo Wu, Xialing Xiong, Xugang Shu, Xuhong Jiang, Xiangjing Qin

**Affiliations:** 1 Institute of Natural Product Chemistry, Zhongkai University of Agriculture and Engineering, Guangzhou, Guangdong, 510225 China; 2 Guangdong Key Laboratory of Marine Materia Medica, South China Sea Institute of Oceanology, Chinese Academy of Sciences(CAS), Guangzhou, 510301 China; 3 Department of Chemical and Biochemical Engineering, Western University, London, Ontario, N6A5B9 Canada

## Abstract

Matrine is a traditional Chinese medicine and botanical pesticide with broad biological activities, including pharmacological and agricultural activities. In present work, two matrine derivatives have been successfully synthesized via introducing indole and cyclohexylamino to 13 position of matrine, respectively, with sophocarpine as starting material, and structurally characterized via infrared spectroscopy(IR), MS, 1 H NMR, 13 C NMR and X-ray crystal diffraction. The results of the *in vitro* biological activity tests showed that these two matrine derivatives exhibited even better activities against human cancer cells Hela229 and insect cell line Sf9 from *Spodoptera frugiperda* (J. E. Smith) than that of parent matrine, suggesting that the heterocyclic or cyclic group can dramatically increase the biological activity of matrine. It is worth to mention that 13-indole-matrine could possibly inhibit the growth of insect cells or human cancer cells by inducing cell apoptosis. The results of the present study provide useful information for further structural modifications of these compounds and for exploring new, potent anti-cancer agents and environment friendly pesticides.

## Introduction

Matrine is a naturally occurring small molecule compound that can be isolated from *Sophora flavescens* (Kushen), *Subprostrata* (Shandougen), and *Alopecuroides* (Kudouzi), being widely distributed in Asia, Oceanica, and the Pacific islands^1,2^. Matrine possesses a broad spectrum of pharmacological activities, such as anti-cancer^3–5^, anti-inflammatory^6^, antiviral^7–9^ antimicrobial^10^, antifibrotic^11^, and immunoinhibitory^12^, etc. In China, matrine is one of the most used derivatives in traditional Chinese medicines^13^ and has been used to treat cancer as well as other diseases such as viral hepatitis, cardiac arrhythmia and skin inflammations, colpitis, and chronic cervicitis^14^. Therefore, considering the good pharmacological effects of matrine, it has a wonderful prospect to develop a practical strategy for the structure modification of matrine to synthesize more derivatives with better pharmacological activities^15^, which would provide a number of excellent candidate compounds for new drug exploration.

Matrine has also been used as an important traditional botanical pesticide^15^, due to its wide range of insecticidal activities, anti-plant virus activity and fungicidal activity in the agricultural field, and being friendly to natural environment^16,17^. More recently, matrine has been used in isolated form or in mixtures with other botanical extracts and synthetic pesticides for the control of termites, aphids, leafhoppers, caterpillars and mites, fungal and bacterial diseases and nematodes in areas of production of vegetables, fruits, flowers and teas in China^15,16,18–20^ as well as in the management of pests of stored grains^21^. Now, matrine has also been commercialized as a broad spectrum insecticide (named as Kudun); however, their insecticidal activities were two orders of magnitude lower than the world’s most popular pesticides discovered by the international pesticide companies in the last ten years. Recently, the synthesis and bioactivity of matrine analogues have been investigated extensively. However, most researches mainly focused on pharmacological activity, as described above. Comparably, the study on its anti-plant virus activity, fungicidal activity, and insecticidal activity for agricultural use of matrine was not much in the literature^22^.

**Figure 1:**
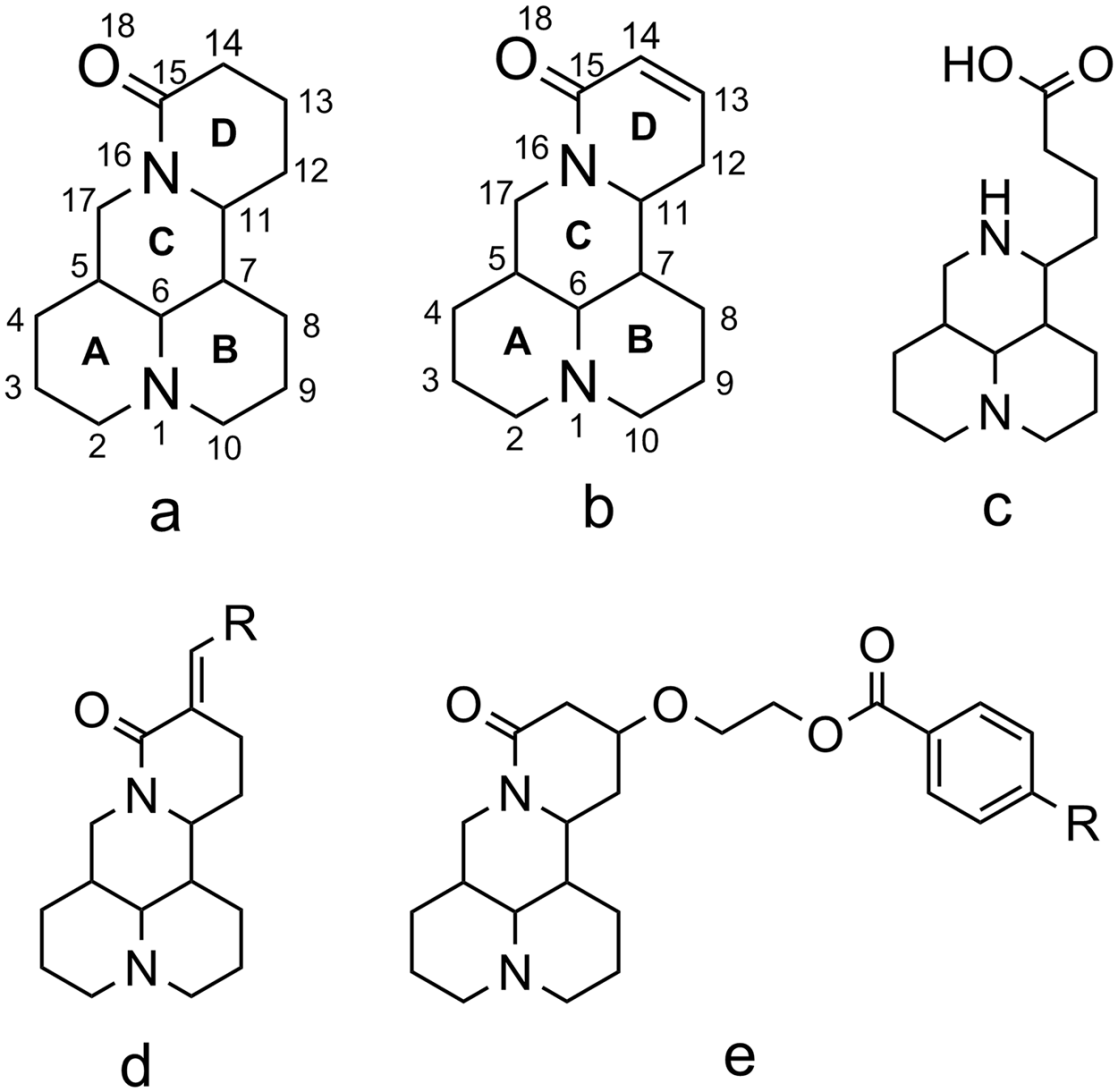
Structures of matrine (**a**), sophocarpine (**b**) and matrine derivatives (**c–e**).

Considering matrine’s good biological activity and its wide use in medicine or agriculture field, it is interesting to develop a general and practical strategy for preparation of more matrine derivatives for the exploration of new anti-cancer drugs or pesticides. Stimulated by above-mentioned promises of matrine, it is useful and desirable for researchers to systematically investigate on modification of matrine. Information on the synthetic methods and various biological activities of matrine derivatives are particularly important and valuable. Given the structural stability of matrine(Fig. 1a), previous studies have mainly focused on the modification of sophocarpine(Fig. 1b), whose structure closely resembles that of matrine. Recently, some matrine derivatives comprising newly introduced substituents to the D-ring at the C13 and C14 positions have shown increased pharmacological activities such as anti-hepatitis B virus (HBV)^7^, anti-inflammatory^23^, and anticancer^24–27^ activities or agricultural use activities such as antiplant virus activity, fungicidal activity^22^, and insecticidal activity^28^. Matsuda *et al*. reported the relationship between molecular structures of quinolizidine alkaloids and their activities against pine wood nematodes and suggested that the cause of nematicidal potency of the cytisine-type structure was the pyridone ring in the molecules^29,30^. In addition, the position and number of double bonds of matrine derivatives also affect their nematicidal activities^31^. Therefore, the work referred to modification on the amide bond or changing position or number of double bonds of matrine might likely effect its biological activity, for example, to open the D-ring of matrine or its derivatives might make them lose almost all antiproliferative activities(Fig. 1c); reduction of carboxyl group of matrine parent core would reduce its anticancer activity and introduction of aromatic acyl group or aromatic vinyl group at C14 position of matrine would improve its anticancer activity(Fig. 1d)^25^. Wang *et al*. synthesized a series of 13-position modified matrine derivatives with sophocarpine as the starting material, and screened their *in vitro* inhibition to the proliferation against HepG2 cells. The results showed that phenyl ring containing matrine derivatives were more active than the alkyl group containing matrine derivatives; Especially, incorporating the phenyl and nitrogen mustard to matrine core could dramatically enhance its inhibition to HepG2(Fig. 1e)^26^. Similar results were also obtained in the structural diversity modification of matrine as a botanical pesticide by Ni *et al*.^22^, where 14-position matrine derivatives with the 15-carbonyl removed were synthesized and systematically evaluated on their antiviral activities against tobacco mosaic virus (TMV), fungicidal activity, and insecticidal activity. The results indicated that compared with parent matrine, when the amido of matrine was reduced to tertiary amine, most derivatives exhibited significantly increased anti-TMV activity, which further demonstrated that 15-carbonyl was adverse to the anti-TMV activity and Chirality of 14-carbon could also influence the activities. Most of these derivatives had a broad spectrum of fungicidal activity and high selectivity on the insecticidal activity against *Culexpipiens pallens*(Diptera:Culicidae) and *Plutellaxylostella*(Linnaeus)^22^. All of 14-formyl-15-aryloxy/methoxymatrines and 14-aryloxymethylidenylmatrine derivatives as pesticidal agents obtained by structural modification of matrine also displayed potent growth inhibitory property against early 3rd-instar larvae of *Mythimna separata* Walker^28^. In the present work, two C-13-position modified matrine derivatives, 13-indole-matrine (1) and 13-cyclohexylamino-matrine(2) have been synthesized by introducing the heterocyclic group, indole and the cyclic group, cyclohexylamino to C-13 of matrine with sophocarpine as starting material, and structurally characterized by infrared spectroscopy(IR), MS, 1 H NMR, 13 C NMR and X-ray crystal diffraction. With the main aim of comparison, we have tested the biological activity of matrine and these two derivatives, including their anti-cancer activity against Hela229 and cytotoxicity against cell line Sf9 from *Spodoptera frugiperda* (J. E. Smith).

## Results

### Chemical synthesis

**Figure 2:**
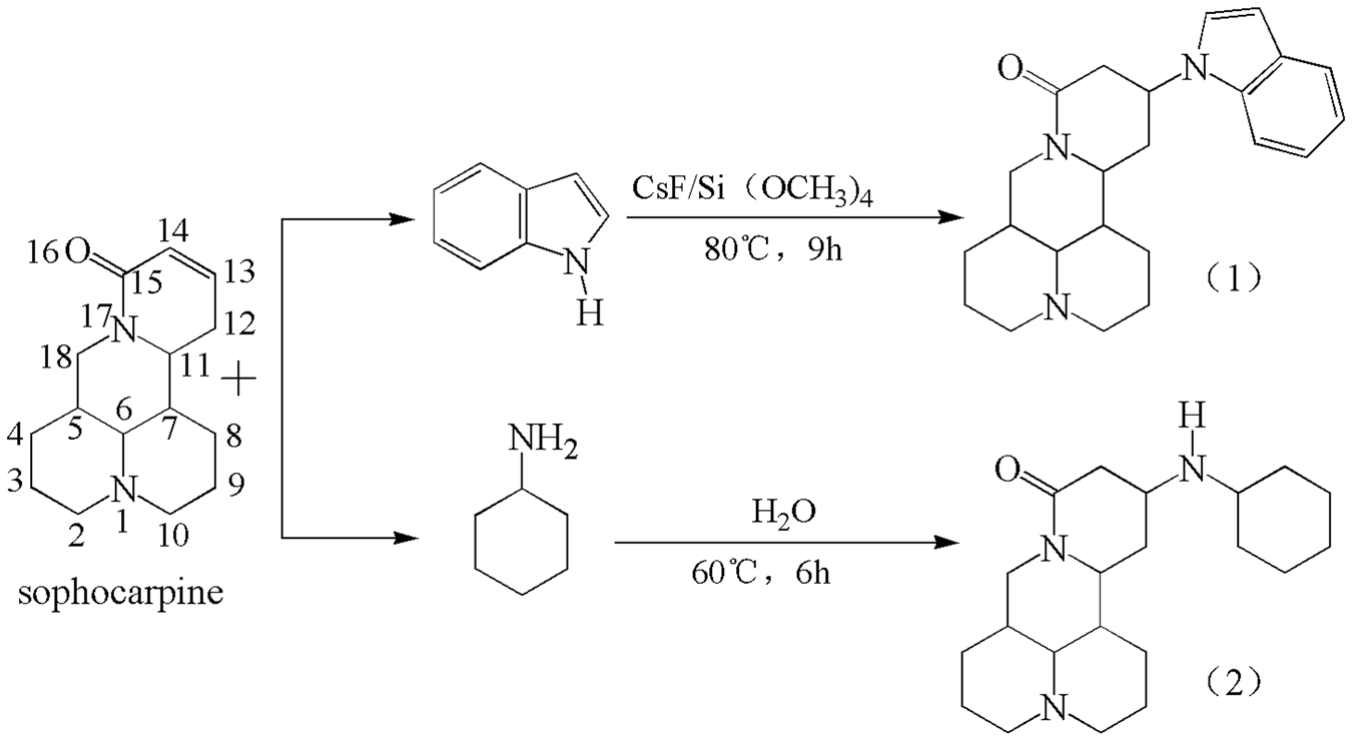
Synthesis of matrine derivatives 1 and 2.

The synthesis of these two matrine derivatives were carried out using the Michael addition reaction (Fig. 2). Matrine derivative 1 was synthesized by reacting sophocarpine and indole (at about 1:2 molar ratio) with cesium chloride (5 mmol) as a catalyst in petroleum ether. The R. Matrine derivative 2 was synthesized by reacting sophocarpine, and cyclohexylamine (about 1:2 molar ratio) in deionized water. These two compounds were crystallized in n-hexane/ethanol (6/1) (for matrine derivative 1) and in petroleum ether (for matrine derivative 2), respectively, whereupon a few colorless, rod-shape crystals suitable for X-ray crystal diffraction analysis were obtained.

### HPLC

**Figure 3:**
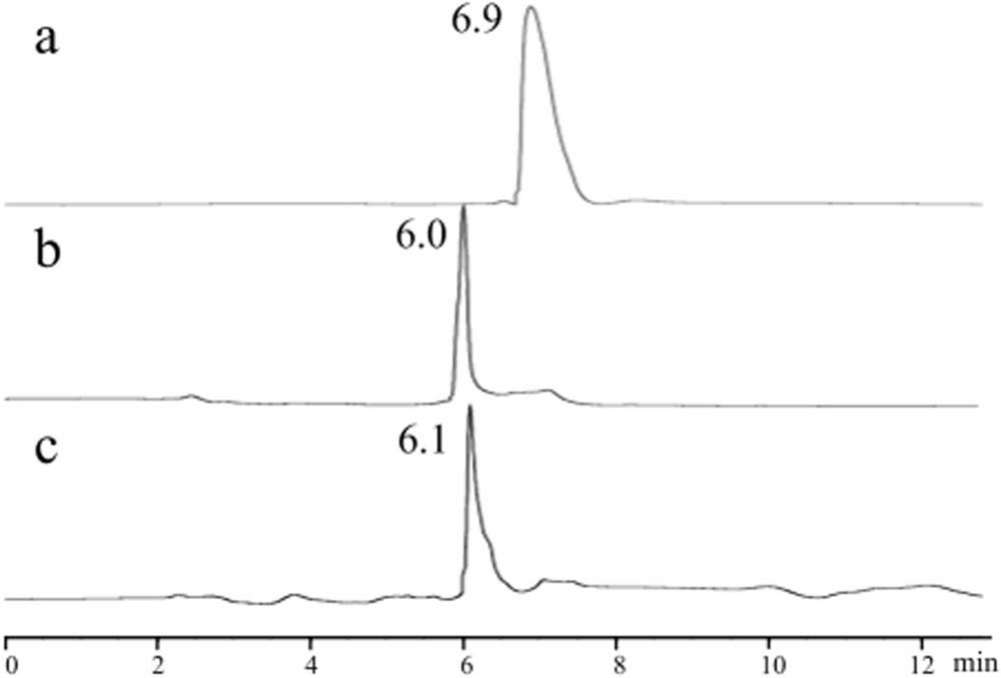
HPLC analysis of sophocarpine (**a**), matrine derivatives 1 (**b**) and 2 (**c**).

HPLC analysis of sophocarpine, matrine derivatives 1 and 2 are shown in Fig. 3a–c.The peaks of sophocarpine, matrine derivatives 1 and 2 were determined to be at 6.9, 6.0, and 6.1 min, respectively and all shows single peak, which indicates these three compounds are pure.

### FT-IR

**Figure 4:**
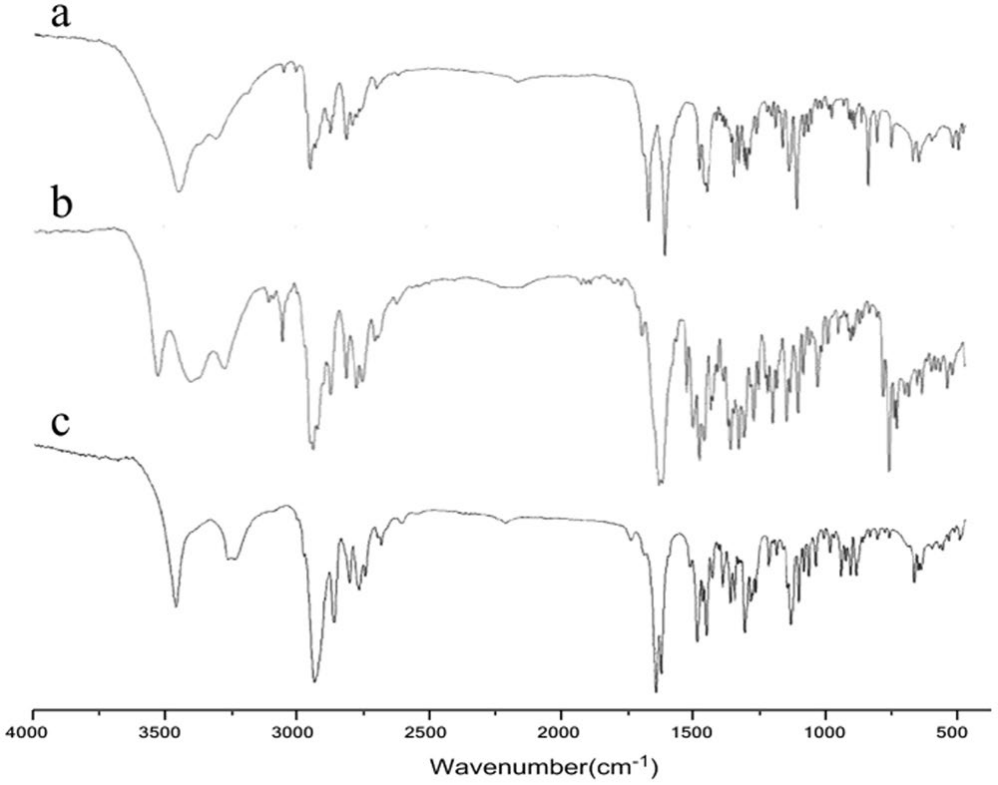
IR spectrum of sophocarpine (**a**), and matrine derivatives 1 (**b**) and 2 (**c**).

IR spectrums of sophocarpine, matrine derivatives 1 and 2 are shown in Fig. 4. As shown by the IR spectrum of sophocarpine, the characteristic bands at around 1660 and 1595 cm^−1^ can be assigned to the ν(C=O) and ν(C=C) absorption of carbonyl  group and double bond (Fig. 4a). From the IR spectrum of the matrine derivative 1 (Fig. 4b), the strong carbonyl stretching band is red-shifted from 1660 cm^−1^ for sophocarpine to 1614 cm^−1^ for matrine derivative 1, due to disappearance of double bond. The three peaks (at 1508, 1486, and 1462 cm^−1^) in range of 1600-1450 cm^−1^ and a sharp peak at 739 cm^−1^ as the characteristic double bond in heterocyclic group are observed in the spectrum, all suggesting that the indole group has been linked to the D ring of matrine skeleton. One can also found from the IR spectrum of matrine derivative 2 (Fig. 4c) that cyclohexylamine group has been introduced successfully to the D ring of matrine skeleton, since the characteristic bands at around 1596 cm^−1^ that can be assigned to ν(C=C) absorption of double bond disappears and the characteristic absorption peak of carbonyl group (1633 cm^−1^) remains. Moreover, a narrow and symmetrical peak at 3457 cm^−1^ and a characteristic band ranging from 1350 to 1100 cm^−1^, attributed to the ν(-NH-) absorption of group -NH-, appear in IR spectrum of the matrine derivative 2.

### LR-ESI-MS

LR-ESI-MS analysis of matrine derivative 1 and 2 is showed in Fig. S1. The molecular formula of matrine derivative 1 and 2 were determined as C_23_H_29_N_3_O and C_21_H_35_N_3_O on the basis of ESI-MS ion peaks at m/z 364.9 ([M + H]^+^, calcd 363.5) and 346.9 ([M + H]^+^, calcd 363.28) respectively.

### NMR

1H NMR and ^13^C NMR spectrums of matrine derivative 1 are shown in Fig. S1. In the 1 H NMR spectrums of Fig. S2A, 6 proton signals of indole unit (H20-δ7.23, H21-δ6.56, H22-δ7.63, H23-δ7.13, H24-δ7.31, H25-δ7.64) are observed, and the other proton signals are consistent with that of previous research^7,25^.The ^13^C NMR spectrum of matrine derivative1 shows 23 carbon signals, attributing to matrine parent core unit (P): C2-C17 (C2-δ 57.15, C3-δ 21.10, C4-δ 27.69, C5-δ 38.18, C6-δ 77.14,C7-δ 42.26, C8-δ 26.63, C9-δ 20.48, C10-δ 57.10, C11-δ 50.88. C12-δ 30.86. C13-δ 63.84, C14-δ 35.78, C15-δ 166.51, C17-δ 46.50 ppm) and indole unit (P): C20–27 (δ 123.67, δ 102.44, 121.30, 119.81, 121.75, 108.96, 135.58, 128.69 ppm) (Fig. 2B). All these results confirm that matrine derivative 1is indeed the target compound and that it is pure.

As show in 1 H NMR and ^13^C NMR spectrums of matrine derivative 2 (Fig. S3), although most of the proton signals in 1 H NMR spectrum overlap, making us hard to identify and analyze (Fig. S3A), 21 carbon signals are acquired distinctly from the ^13^C NMR spectrum. All of them attribute to matrine parent core unit (P): C2-C17 (C2-δ57.20, C3-δ21.12, C4-δ27.69, C5-δ39.13, C6-δ77.17,C7-δ42.39, C8-δ26.58, C9-δ20.66, C10-δ57.16, C11-δ50.43. C12-δ31.63. C13-δ63.89, C14-δ35.58, C15-δ167.79, C17-δ44.72ppm) and cyclohexylamino unit (P): C20–25 (C20-δ53.29, C21-δ34.02, C22-δ25.02, C23-δ 26.01, C24-δ24.98, C25-δ33.81 ppm) (Fig. S3B). All these fully confirm that the product was indeed the target compound. Meanwhile, the result also shows that matrine derivative 2 is pure.

### XRD

**Figure 5:**
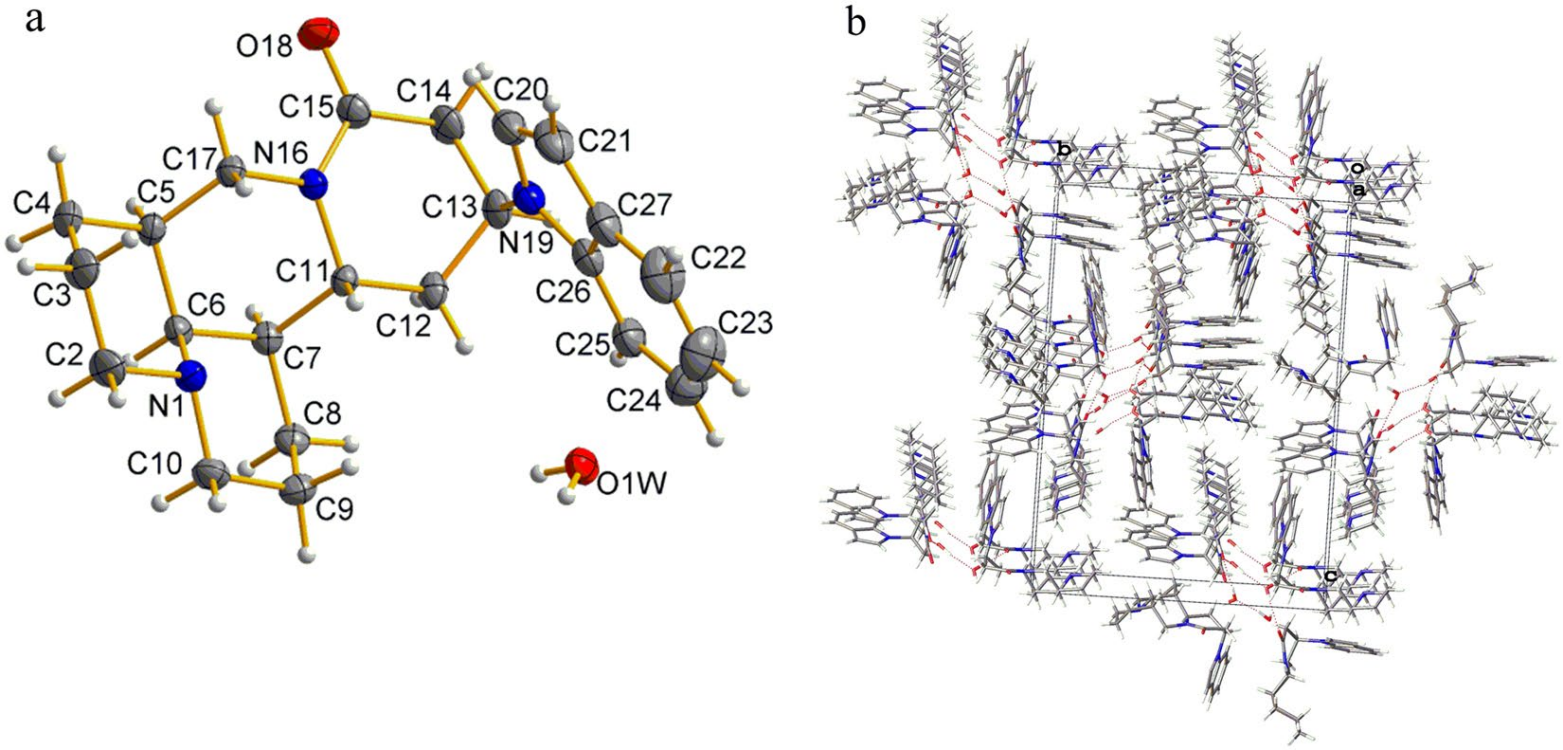
Crystal structure of matrine derivative 1 (**a**) and packing of its molecule in a unit cell (**b**).

Some key crystallographic data of matrine derivative 1 are provided in Tables 1 and 2. Indole group is linked to C13 position of matrine, forming a bond (C13-N19) with bond length 1.468(17) Å (Fig. 5a). Meanwhile, the bond length of C12-C13 [1.523(18) Å], C13-C14 [1.517(19) Å] and C14-C15 [1.512(18) Å] in the D ring of matrine derivative 1 is somewhat longer in comparison with that of sophocarpine, being 1.4925(16) Å, 1.323(18) Å and 1.478 Å, respectively. However, the bond length of C11-C12 [1.519(16) Å] in matrine derivative 1 does not change significantly compared to that of sophocarpine, being 1.529(16) Å. The bond angle of C12-C13-C14 and C13-C14-C15 in D ring of matrine derivative 1 with a value of 107.45(11)° and 113.80(11)°, respectively, decreases obviously if compared to that of sophocarpine[120.73(10)° and 121.78(11)°, respectively], while the N16-C15-C14 angle remains nearly the same, being 117.93(11)°. All these indicate that the introduction of indole group to C13 of D ring mainly affects the length of the nearby bonds, the angle and the configuration of C13. The bonds (C14-C13) and (C12-C13) form an angle of 111.99(11)° and 112.61(10)°, respectively with the bond (N19-C13). Meanwhile, the bond (N19-C13) forms an angle of 128.63(12)° with (N19-C20). In addition, the molecules are packed in the crystal structure without any hydrogen bonds, and the benzene rings of the molecules in the crystal structure of the matrine derivative 1 show no *π-π* interaction (Fig. 5b).

**Table 1:** Crystal and structure refinement data of matrine derivatives 1–2.

Matrine derivative	1	2
Empirical formula	C_23_H_29_N_3_O	C_21_H_35_N_3_O
Formula weight	363.5	345.53
Temperature (K)	293(2)K	273(2)K
Wavelength (A°)	1.54178	1.54178
Crystal system	orthorhombic	orthorhombic
Space group	P21	P21
*a* (Å)	8.62130(10)	12.38460(10)
*b*(Å)	18.25770(10)	31.5861(3)
*c*(Å)	25.6873(2)	5.292
α(°)	90	90
β(°)	90	117.825(4)
γ(°)	90	90
V (A° ^3^)	4043.31(6)	2070.13(3)
Z	8	1
Dc (g cm^−3^)	1.253	1.167
Mu (Mo K_α_) (mm)	0.636	0.587
F (000)	1648	800
Crystal size (mm)	0.36 × 0.30 × 0.20	0.28 × 0.20 × 0.1
Reflections collected	38567	15404
Independent reflection	8120[R(int) = 0.0391]	4131[R(int) = 0.0351]
Goodness-of-fit on *F*^2^	1.001	1.066
Final *R* indices [*I* > 2σ(*I*)]	*R*_1_ = 0.0305, *ωR*_2_ = 0.0807	*R*_1_ = 0.0380, *ωR*_2_ = 0.1026
*R* indices (all data)	*R*_1_ = 0.0318, *ωR*_2_ = 0.0793	*R*_1_ = 0.0388, *ωR*_2_ = 0.1019

**Table 2:** Selected bond lengths (Å) and bond angles (°) of matrine derivatives 1–2.

Sophocarpine			
C12-C13	1.4925(16)	C12-C13-C14	120.73(10)
C13-C14	1.3229(18)	C13-C14-C15	121.78(11)
C11-C12	1.529(16)	N16-C15-C14	117.84(10)
C14-C15	1.478(16)		
**1**			
C12-C13	1.5225(18)	C12-C13-C14	107.45(11)
C13-C14	1.517(19)	C13-C14-C15	113.80(11)
C11-C12	1.519(16)	N16-C15-C14	117.93(11)
C14-C15	1.512(18)	N19-C13-C14	111.99(11)
N19-C13	1.468(17)	N19-C13-C12	112.61(10)
		C20-N19-C13	128.63(12)
**2**			
C12-C13	1.526(2)	C12-C13-C14	108.18(12)
C13-C14	1.529(18)	C13-C14-C15	116.58(11)
C11-C12	1.525(18)	N16-C15-C14	118.47(12)
C14-C15	1.512(19)	N19-C13-C14	107.87(11)
N19-C13	1.476(16)	N19-C13-C12	113.60(12)
N19-C20	1.475(17)	C20-N19-C13	116.43(11)

**Figure 6:**
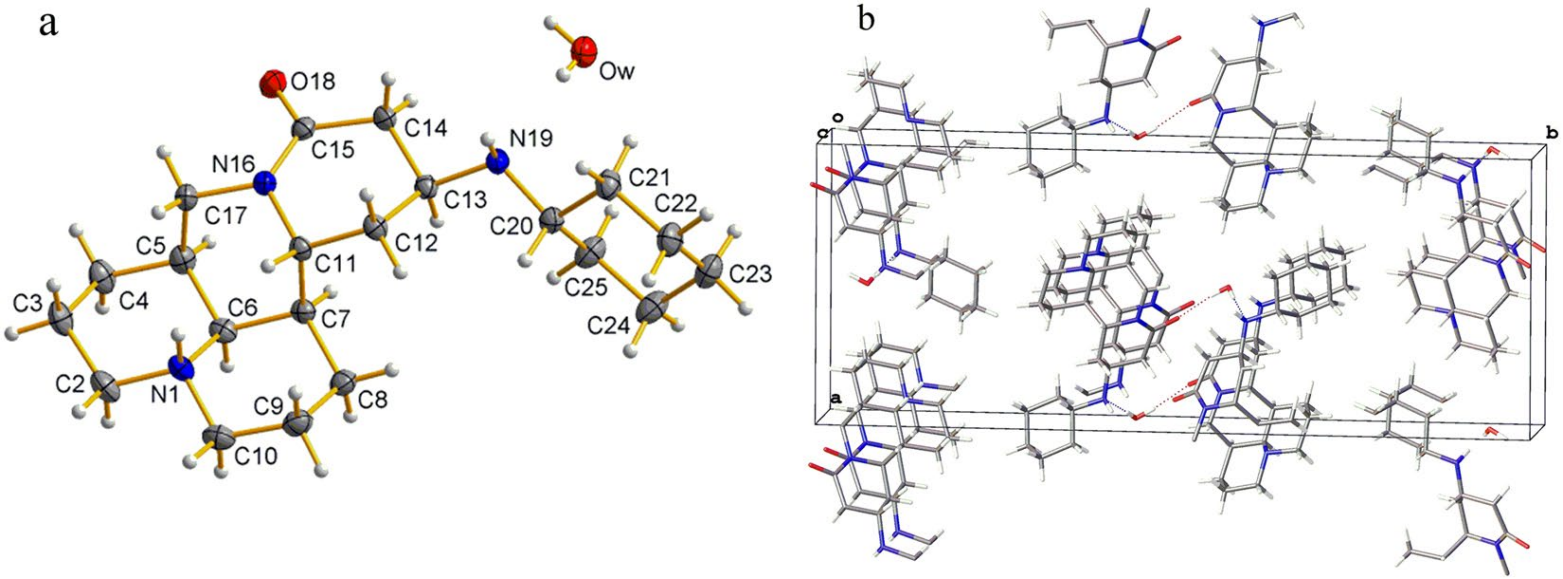
Crystal structure of matrine derivative 2 (**a**) and packing of its molecule in a unit cell (**b**).

In accordance to the key crystallographic data of matrine derivative 2, as also shown in Tables 1 and 2, cyclohexylamine group was successfully introduced to C13 position of matrine (Fig. 6a), and forms a branched chain (C13-N19-C20) linking the matrine skeleton and cyclic group, with the bond length of 1.476(16) Å and 1.475(17) Å for C13-N19 and N19-C20, respectively. The bond length of C12-C13 [1.526(2) Å], C13-C14 [1.529(18) Å] and C14-C15 [1.512(19) Å] in D ring of matrine derivative 2 is somewhat longer in comparison with that of sophocarpine, 1.4925(16) Å, 1.3229(18) Å and 1.478 Å, respectively, similar as that of matrine derivative 1 discussed previously. However, the bond length of C11-C12 [1.525(18) Å] in matrine derivative 2 does not change significantly compared to that of sophocarpine (1.529(16) Å). The bond angle of C12-C13-C14 [108.18(12)°] and C13-C14-C15 [116.58(11)°] in D ring of matrine derivative 2 decreases obviously if compared to that of sophocarpine [120.73(10)° and 121.78(11)°, respectively]. Nevertheless, the N16-C15-C14 angle increases slightly to be 118.47(12)°. All these indicate that the introduction of cyclohexylamine group to C13 of D ring mainly influences the length of the nearby bonds, the angle and the configuration at C13, as observed and discussed previously for matrine derivative 1. The molecules are packed in the crystal structure without any hydrogen bonds and the benzene rings of the matrine derivative 2 molecules in the crystal structure show no π-π interaction (Fig. 6b).

### Cytotoxicity assay against insect cell

**Figure 7:**
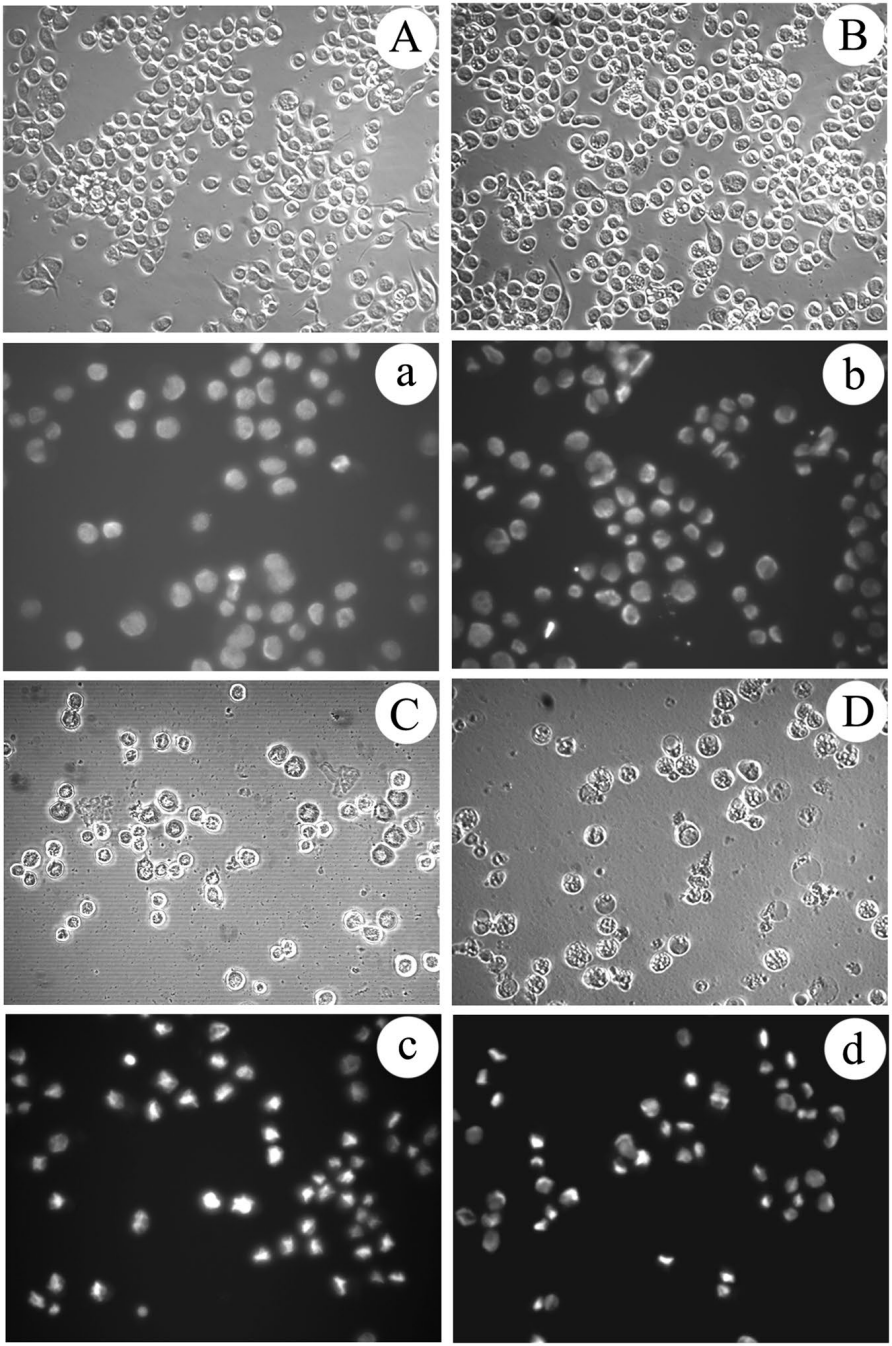
Analysis of proliferation and cell morphological change in sf9 cells treated with matrine derivatives 1 and 2 for 48 h. Photographs marked with capital letters were obtained from IPCM (inverted phase contrast microscopy), with small letters from FM (fluorescence microscopy); magnification was 200X . (**A**) Morphological characteristics of cell in control; (a) Morphological characteristics of cell nucleus in control; (**B**) Morphological changes of cell induced by matrine; (b) Nucleus morphological changes of cell induced by matrine; (**C**) Morphological changes of cell induced by matrine derivative 1; (c) Nucleus morphological changes of cell induced by matrine derivative 1; (**D**) Morphological changes of cell induced by matrine derivative 2; (d) Nucleus morphological changes of cell induced by matrine derivative 2.

**Figure 8:**
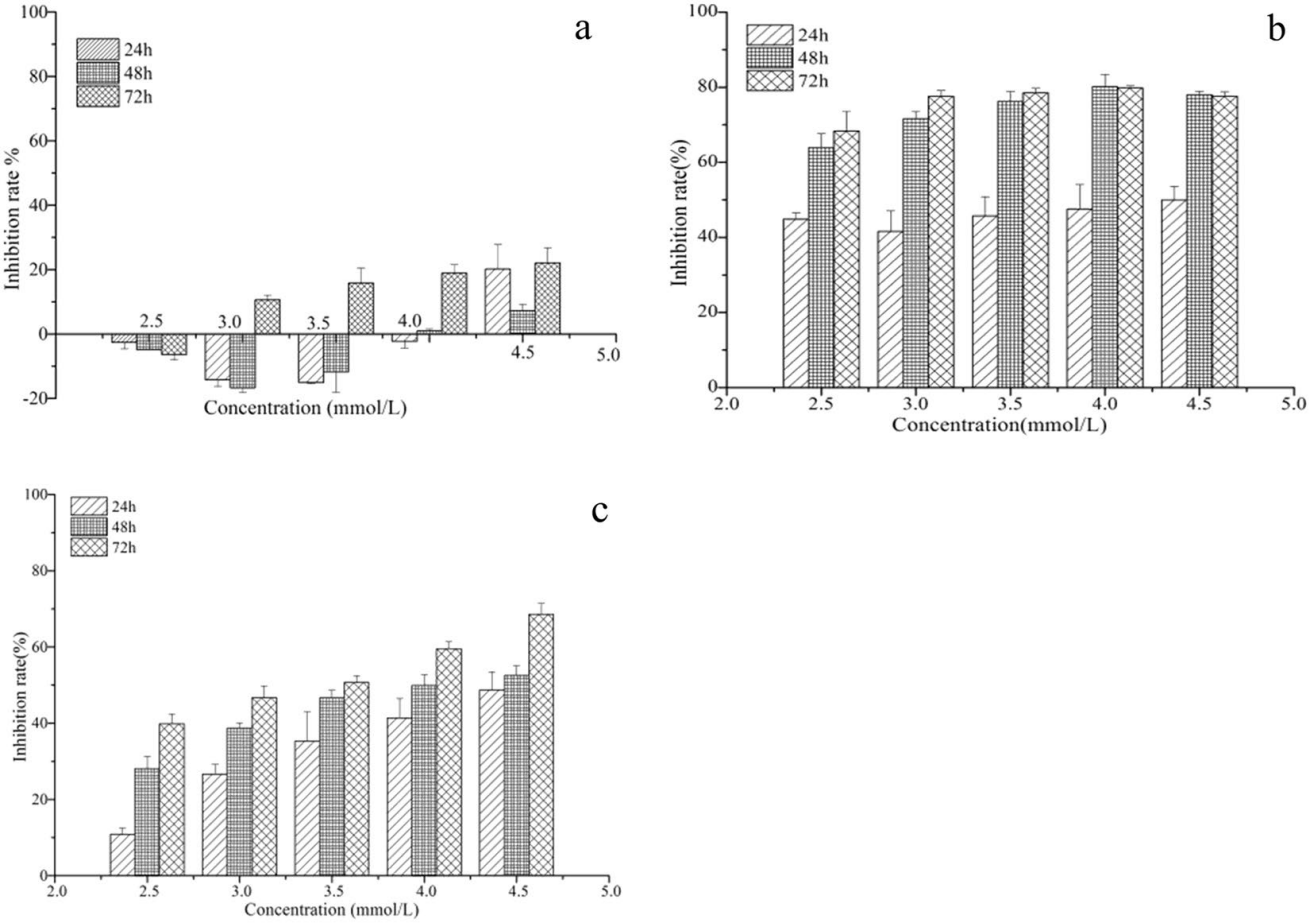
Inhibition rate of Sf9 cells after treatment with matrine (**a**), matrine derivatives 1 (**b**) and 2 (**c**). All data represented are mean ± S.E.M. from three independent experiments.

*In vitro* antiproliferative activities of the obtained matrine derivatives as well as the parent matrine were evaluated by different assays against the human cancer cell lines Hela229 and the insect cell line Sf9 from *Spodoptera frugiperda* (J. E. Smith). As shown in Fig. 4, cellular morphological changes of Sf9 cells were observed under IPCM (inverted phase contrast microscopy); cells in control group and in the matrine treated group with concentration of 2.5 mmol/L are presented in round shape and are adhered well (Fig. 7A,B). After treated with matrine derivative 1 with the same concentration (i.e., 2.5 mmoL/L) for 48 h, few normal cells, a large amount of apoptosis bodies and floating cells were observed (Fig. 7C); according to results of nucleus morphological measurement by staining with 2.3 4,6-Diamidino-2-phenylindole(DAPI), the cell nucleus of the matrine derivative 1 treated groups are condensed, erose, swelled or paged (Fig. 7c), whereas the control and matrine treated groups still held complete and circular nucleus (Fig. 7a,b), all the above indicate that the matrine derivative 1 induced the Sf9 cells apoptosis. And the above described morphological changes of apoptosis are generally consistent with our previous study on azadirachtin inducing Sf9 cells apoptosis^32^. Interestingly, the matrine derivative 2 also exhibited notable inhibition activity on Sf9 cells, but the cellular morphological changes seemed to be different from that treated by matrine derivative 1. As one can observe in Fig. 7D and d, although the number of cells also decreased sharply after treatment for 48 h with 2.5 mmol/L of matrine derivative 2, few floating cells and apoptosis bodies were observed and most of the adhered cells showed characteristic vacuolation *in situ* or nucleus condensation, finally disintegrated and disappeared. Furthermore, the cell viability assessed by 3-(4,5-dimethylthiazole-2yl)-2,5-diphenyl (MTT) test showed that when Sf9 cells were in matrine for 24, 48, and 72 h, with a concentration below 4, 3.5, 2.5 mmol/L, respectively, the growth of Sf9 cells was indeed improved slightly, but was inhibited at a higher concentration, with an inhibition rate only below 20% at a concentration of 3–4.5 mmol/L (Fig. 8a), and the IC50 was 7.68, 10.07, and 6.40 mmol/L of matrine treatment for 24, 48, 72 h, respectively (Table 3). In contrast, both matrine derivatives 1 and 2 exhibited significant antiproliferative activities against Sf9 cells in a wide concentration range of 2.5–4.5 mmol/L; matrine derivative 2 showed notable time and concentration dependence, while matrine derivative 1 showed only time-dependence at a concentration less than 3.5 mmol/L; when the cells were treated with 3 mmol/L matrine derivatives 1 and 2, respectively for 48 h, the inhibition rate for these two matrine derivatives reached 81.62% and 46.63%, respectively, much higher than that of matrine (1.02%) (Fig. 8b,c), displaying their stronger inhibition activities against Sf9 cells compared to that of matrine. All these results indicated that the introduction of indole or cyclohexylamine group to C13 of D ring of matrine skeleton significantly improves its antiproliferative activity against insect cell lines.

**Table 3:** IC50 of matrine derivatives 1 and 2 against Sf9 cells.

Compounds	IC50 (mmol/L)		
	24 h	48 h	72 h
Sophocarpine	5.59 ± 1.6	5.46 ± 0.87	5.30 ± 0.59
Matrine	7.68 ± 0.91	10.07 ± 3.1	6.40 ± 1.8
Matrine derivatives 1	5.08 ± 0.26	0.12 ± 0.03	0.07 ± 0.01
Matrine derivatives 2	4.46 ± 1.4	4.07 ± 1.3	3.28 ± 0.76

### Anticancer activity assay

**Figure 9:**
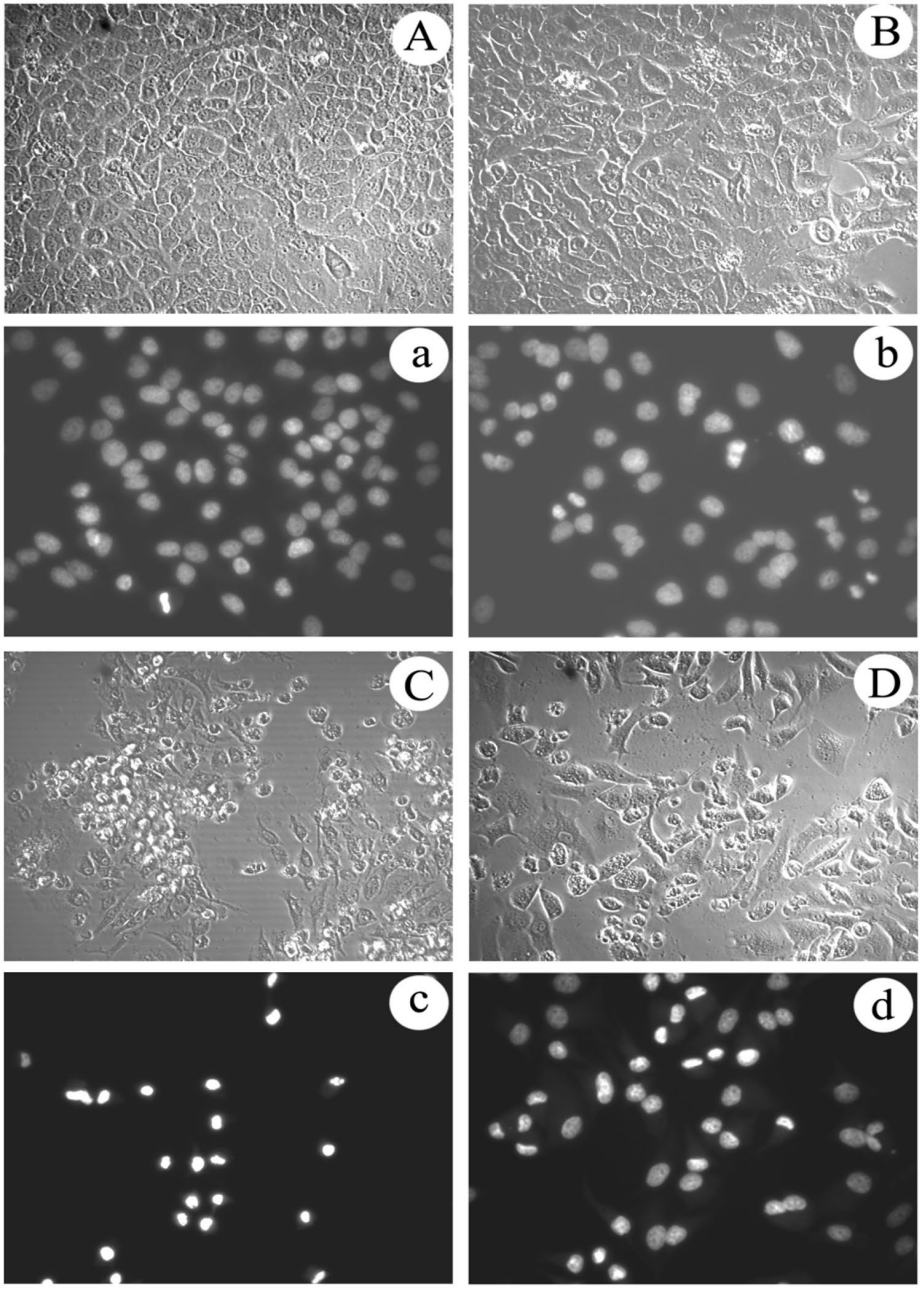
Analysis of proliferation and cell morphological change in Hela229 cells treated with matrine derivatives 1 and 2 for 48 h. Photographs marked with capital letters were obtained from IPCM (inverted phase contrast microscopy), with small letters from FM (fluorescence microscopy); magnification was 200X . (**A**) Morphological characteristics of cell in control; (a) Morphological characteristics of cell nucleus in control; (**B**) Morphological changes of cell induced by matrine; (b) Nucleus morphological changes of cell induced by matrine; (**C**) Morphological changes of cell induced by matrine derivative 1; (c) Nucleus morphological changes of cell induced by matrine derivative 1; (**D**) Morphological changes of cell induced by matrine derivative 2; (d) Nucleus morphological changes of cell induced by matrine derivative 2.

In order to demonstrate the biological activity of these two compounds for human cancer cells, *in vitro* antiproliferative activities assays against the human cancer cell lines Hela229 were carried out subsequently. Cellular morphological changes were observed under IPCM, and the nucleus morphological characteristic change was measured by staining with DAPI. The results in IPCM and DAPI staining assays showed that cells in both the control and matrine treated groups (1.4 mmol/L) grew well and adhered firmly, presenting in fusiform or polygon morphology with complete (Fig. 9A,B) and circular nucleus (Fig. 9a,b), while the cells treated with 0.64 mmol/L matrine derivative 1 shrank significantly with a large number of apoptosis bodies and floating cells observed (Fig. 9C) and the nucleus presented erose, swelled, shrank or paged shape (Fig. 9c), suggesting that matrine derivative 1 induced the cancer cell apoptosis. Similarly, the matrine derivative 2 also exhibited significant inhibition activity against Hela229 cell. When treated with 1.0 mmol/L matrine derivative 2 for 48 h, the cell number decreased dramatically, with few floating cells and some adhered cells observed compared with the matrine derivative 1-treated group. Moreover, among the adhered cells, most of them were not shrunk but swelled instead (Fig. 9D), a characteristics much different from that of the matrine derivative 1-treated group. In addiction, many adhered cells contain disintegrated, swelled, shrunk or vacuolar nucleus (Fig. 9D,d) similar to the matrine derivative 2-treated insect cells as described previously.

**Figure 10:**
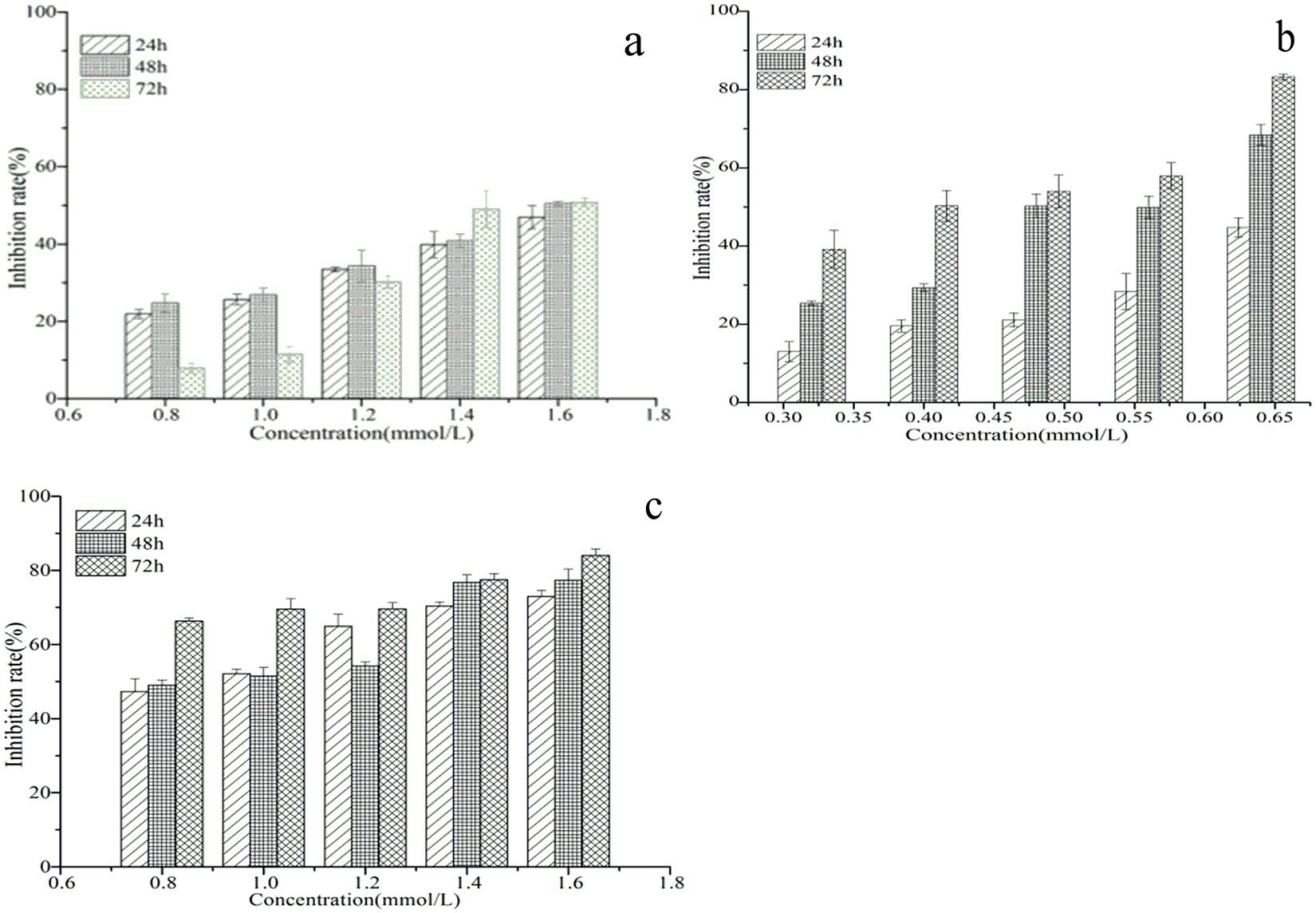
Inhibition rate of Hela229 cells after treatment with matrine (**a**), matrine derivative 1 (**b**) and matrine derivative 2 (**c**). All data represented are mean ± S.E.M. from three independent experiments.

Similarly, MTT assay was performed to measure the anticancer activity of matrine derivative 2, compared with its parent martine compound, against Hela229. The results indicated that in the presence of 0.8–1.6 mmol/L parent matrine for 24, 48, and 72 h, respectively, the growth of Hela229 cells was inhibited to some extent, depending on the treatment time and concentration. The inhibition rate ranged from 25.79–57.93% (Fig. 10a) and IC50 was 1.71, 1.64 and 1.53 for those three durations of treatment, respectively (Table 4), suggesting that the parent compound - matrine did exhibit a certain anti-cancer activity against Hela229 cells, as also reported in some previous studies^3–5^. In contrast, the cells incubated in matrine derivative 1 at a much lower concentration (0.32–0.64 mmol/L) were inhibited significantly, depending on concentration and time of the treatment; the inhibition rate at 0.64 mmol/L (48 h) reached 68.41%, still much higher than that of 0.8 mmol/L matrine treatment (24.79%) (Fig. 10b); the IC50 value was 0.52, again much lower than that of the matrine treatment (IC50 = 1.64), which indicats that the anti-cancer activity of the matrine derivative 1 is about 3 folds higher than that of matrine (Table 4). We obtained similar results while evaluating the anti-cancer activity of matrine derivative 2 in MTT assay. When the cells were incubated in matrine derivative 2 with the same treatment conditions (concentration and time) as the above used for matrine, the cells growth was inhibited significantly, with an inhibition rate reaching 47.04–84.03% within the concentration range of 0.8–1.6 mmol/L (Fig. 10c), much higher than that of the matrine treatment; the IC50 was 0.87,0.91 and 0.12 for those three treatment durations, respectively, also much lower than those of the matrine treatment (Table 4).

**Table 4:** IC50 of matrine derivatives 1 and 2 against Hela229 cells.

Compounds	IC50 (mmol/L)		
	24 h	48 h	72 h
Sophocarpine	1.69 ± 0.30	1.64 ± 0.24	1.50 ± 0.80
Matrine	1.71 ± 0.19	1.64 ± 0.54	1.53 ± 0.32
Matrine derivatives 1	0.75 ± 0.10	0.52 ± 0.06	0.42 ± 0.12
Matrine derivatives 2	0.87 ± 0.26	0.91 ± 0.17	0.12 ± 0.03

## Discussion

As described earlier in the present study, the results clearly showed that introduction of indole or cyclohexylamine group to 13-position of D ring of matrine skeleton could greatly improve its antiproliferative activities against both insect cells and human cancer cells. Especially, the matrine derivative 1 (with indole group) indicated much stronger growth inhibition activity than matrine derivative 2 (with cyclohexylamine group), resulting in much lower IC50 values (0.12 mmol/L for the insect cell and 0.52 mmol/L for the human cancer cell tested). Our results are also in a good agreement with the previous research^26^. Considering the structural features of matrine derivative 1, one may deduce that the introduction of indole to matrine skeleton could improve its permeability into cell membrane and then influence its bioactivity, hence resulting in increased lipophilicity of this derivative^12,33,34^. This explanation could also be applicable for matrine derivative 2, even though its lipophilicity is relatively lower than that of matrine derivative 1, but much higher than that of the parent matrine (Table 4).

As discussed previously, although the inhibition activities of matrine derivatives 1and 2 against two kinds of cells were both stronger than that of matrine, matrine derivative 1 exhibited significantly stronger inhibition activity against insect cells compared to the human cells for 48 h treatment, with the IC50 value for the incent cells 5-fold lower than that for Hela229 cells. However, opposite performance was observed for the matrine derivative 2 for 48 h treatment, as it exhibited much stronger inhibition activity against the human cancer cells than that against the insect cells, with the IC50 value for the human cancer cells 4-fold lower than that of insect cells under the same conditions. As such, the antiproliferative mechanisms of matrine derivatives 1 and 2 seem to be different. As discussed previously, the matrine derivative 1 possibly inhibited the growth of insect cells or human cancer cells by inducing cell apoptosis, whereas the inhibited mechanism of matrine derivative 2 on these cell lines is still not clear, so further investigation is needed.

## Conclusion

In summary, two matrine derivatives have been successfully synthesized by introducing indole and cyclohexylamino groups to 13 position of matrine, respectively, and structurally characterized via IR, MS, 1 H NMR, 13 C NMR and X-ray crystal diffraction analyses. The preliminary *in vitro* biological activity tests indicated that comparing to the parent matrine, these two matrine derivatives exhibited stronger cytotoxic activities against both human cancer cells Hela229 and inset cell line Sf9, suggesting the contribution of heterocyclic or cyclic group to the increasing of the biological activities of matrine. Further biological evaluations, such as *in vivo* biological activity tests in mammals or insects, and fungicidal activity, are ongoing in our laboratory.

## Materials and Methods

### Materials

Sophocarpine (purity99%) was purchased from Baoji Fangsheng Biological development Co., Ltd. 2-Aminopyridine, indole, cesium chloride,tetramethoxysilane and cyclohexylamine were purchased from Shanghai Aladdin Bio-Chem Technology Co., Ltd. Column chromatography was purchased from Qingdao Haiyang Chamical Co., Ltd.The other reagents are all of analytical grade or HPLC grade according to the indeed necessary.

### Analytical methods

IR spectra were recorded on a PerkinElmer Spectrum 100 with KBr disks. 1 H NMR and 13 C NMR spectra 1 H and 13 C NMR spectra were recorded at 25  C with an Avance 500 MHz spectrometer(Bruker). The LR-ESI-MS analysis was performed using a Bruker amaZon SL. HPLC analysis was carried out using Agilent HPLC1200, with main chromatograph conditions, including chromatograph column CNW Athena C18-WP (4.6 mm × 250 mm, 5 µm); mobile phase 10:80:10 (v/v) ethanol/ratio of acetonitrile/KH_2_PO_4_ (ф = 0.3%) with flow rate 1.0 mL·min^−1^; detection wavelength 210 nm.

### Synthesis of matrine derivative 1

The synthesis of 13-indole-matrine was carried out using the Michael addition reaction. A mixture of sophocarpine (1.3 g, 5.0 mmol), indole (0.9 g, 8 mmol) and cesium chloride (0.8 g, 5 mmol) was dissolved in 12 mL petroleum ether, and stirred vigorously at 80 °C for reaction with the conditions of nitrogen protection and reflux condensation. After 5 min, tetramethoxysilane (0.5 mL, 2.5 mmol) was added dropwise with continued stirring and reacting for 9 h. The crude reaction mixture was collected after adding 10 mL of dichloromethane and filtrated with filter tissue. A concentrated brown solution was obtained after removing dichloromethane with a rotary evaporator. The concentrated resulting solution was purified by the silica gel column chromatography eluting with ethyl acetate/ethanol = 10/1 (v/v). The title compound was crystallized from n-hexane/ethanol (6/1, v/v), whereupon a few colorless, rod-shape crystals suitable for X-ray diffraction analysis were obtained. The percent yield was 70.2% and 0.913 g product was got in this reaction.

### Synthesis of matrine derivative2

The synthesis of 13- cyclohexylamino-matrine was carried out using the Michael addition reaction. A mixture of sophocarpine (0.62 g, 0.25 mmol), and cyclohexylamine(0.50 mL) was dissolved in 4 mL deionized water, and stirred vigorously at 60 °C for reaction. After 6 h, the crude reaction mixture was collected and concentrated with a rotary evaporator. The concentrated resulting solution was purified with the silica gel column chromatography eluting with ethanol/ethyl acetate = 1/8 (v/v). The purified compound was first dissolved in petroleum ether under 50–60 °C, and then cooled naturally, at last the title compound was crystallized from petroleum ether, whereupon a few colorless, rod-shape crystals suitable for X-ray diffraction analysis were obtained. The compound was characterized via infrared spectroscopy and X-ray diffractometer.The percent yield was 43.02% and 0.374 g product was got in this reaction.

### Cell line and culture conditions

*Spodoptera frugiperda* Sf9 cells were cultivated at 27 °C C in 25 cm^2^ flasks in 3 mL Grace’s insect cell culture medium (Gibco, USA) containing 10% fetal bovine serum (FBS), 0.3% yeast extract, 0.3% lactalbumin hydrolysate and 0.3% peptone. Hela229 cells were purchased from Cell Bank of Chinese Academy of Sciences and were incubated with Dulbecco’s Modified Eagle’s Medium (Gibco®; Life Technologies, Carlsbad, CA, USA) and supplemented with 10% fetal bovine serum (Gibco®; Life Technologies), 100 U/mL of penicillin, and 100 mg/mL of streptomycin at 37 °C with 5% CO_2_. All cells were seeded and planked in 35 mm cultural plates for 24 h when the cells were at the optimum conditions for the following treatment.

### Evaluation of cells viability by 3-(4,5-dimethylthiazole-2yl)-2,5-diphenyl (MTT) assays

An MTT assay was performed to evaluate cytotoxicity against Sf9 cells and anti-cancer activity against Hela229 cells of these two matrine derivatives. Sf9 Cells in good condition were selected and incubated for 24 h at 27 °C in the 96-well plates with 100 uL cell suspensions in each hole. Moderate quantity of matrine derivative (different final concentrations preset) was added into the cell suspensions. 0.1% DMSO was used as the control. After different treatment durations (24, 24, 48 and 72 h, respectively), 10 uL freshly prepared MTT reagent (5 mg/mL) was added and the plates were incubated in darkness for 4 h at 27◦C. Then the medium was discarded, and then 150 uL fresh DMSO was added to each well to dissolve the formazan crystal by orbital shaking in darkness for 15 min. The absorbance was measured at 490 nm with a microplate reader (BioTek, USA). Cell viability was calculated by the following equation: Cell viability (%) = (1-OD treatment)/(OD control) × 100%. The resulting data were analyzed using Origin 7.5 software (OriginLab, USA). All experiments were performed in parallel and in triplicate. For data analysis, IC50 values were obtained by using Origin 7.5 (OriginLab, USA). MTT assay for Hela229 was similar with that of Sf9 cell, except for the cells incubated at 37 °C under a 5% CO_2_ atmosphere.

### 2.3 4,6-Diamidino-2-phenylindole (DAPI) staining analysis. Cells were seeded in

12-well plates, and exposed to matrine derivative 1or 2 at a preset concentration for 48 h, and then the nutrient supernatant was discarded. The uncovered cells were fixed in 4% paraformaldehyde for 15 min and washed with PBS twice. Finally, the cells were stained in DAPI dye liquor (Southern Biotech Company, USA) at the final concentration of 1 mg/L for 15 min and washed in PBS once again. The samples were observed and photographed by fluorescence microscope (FM) (Olympus BX51, Olympus, Japan).

### Statistical analysis

The data are presented as the mean ± S.E.M of three independent experiments. Statistical analyses were performed using SPSS 18.0 (SPSS, Inc., USA). The graphs were drawn with Origin 7.5 (Origin Lab, USA).

## Electronic supplementary material

Supplementary Information
