# Supplementary material for: Synthesis, characterization and in vitro biological evaluation of two matrine derivatives
Source: Sci Rep. 2018 Oct 24;8:15686. doi: 10.1038/s41598-018-33908-8 (PMC6200782; doi:10.1038/s41598-018-33908-8)
Supplement: Supplementary file 1 — Supplementary Information [file 41598_2018_33908_MOESM1_ESM.pdf]

Synthesis, characterization and in vitro biological evaluation of two matrine derivatives

Xingan Cheng[1],Jingmin,Ye[1], Huiqing He[1], Zhanmei Liu[1], Chunbao (Charles) Xu[3], Bo Wu[1], Xialing, Xiong, Xugang Shu[1], Xuhong Jiang[1]*, Xiangjing Qin[2]*

[1]Institute of Natural Product Chemistry, Zhongkai University of Agriculture and Engineering, Guangzhou, Guangdong 510225, China

[2]Guangdong Key Laboratory of Marine Materia Medica,South China Sea Institute of Oceanology , Chinese Academy of Sciences(CAS), Guangzhou 510301, China

[3]Department of Chemical and Biochemical Engineering, Western University, London, Ontario N6A5B9, Canada

*Corresponding authors. E-mail address:jiangxh69@163.com (X.H.Jiang) ;xj2005qin@126.com(X.J.Qin)

Supporting information:

Supplementary Figure S1-S3.

Supplementary materials

Figure S1. LR-ESI-MS analysis of matrine derivative 1(a) and 2(b).

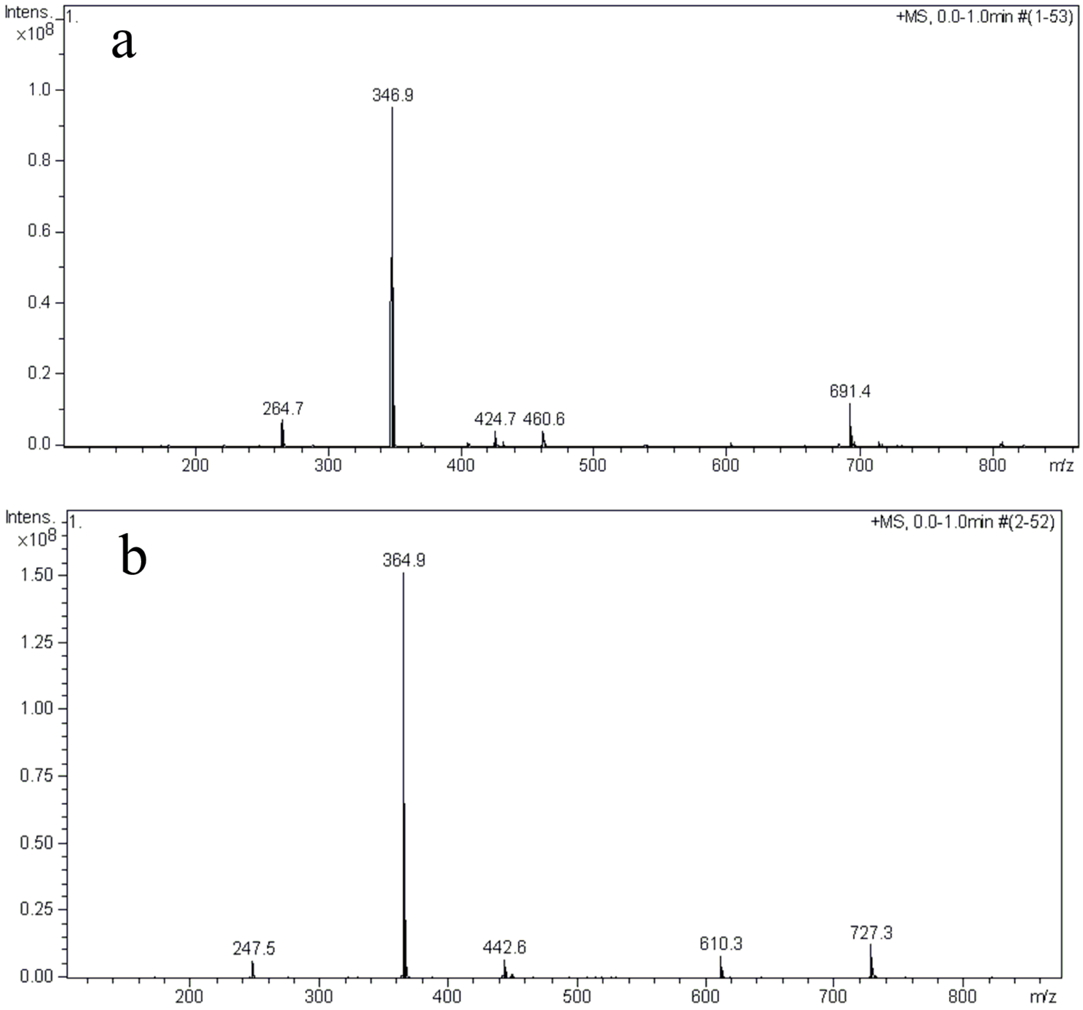

Figure S2. ^{1}H NMR (A) and ^{13}C NMR (B) spectrums of matrine derivative 1 in $CDCl_3$

A

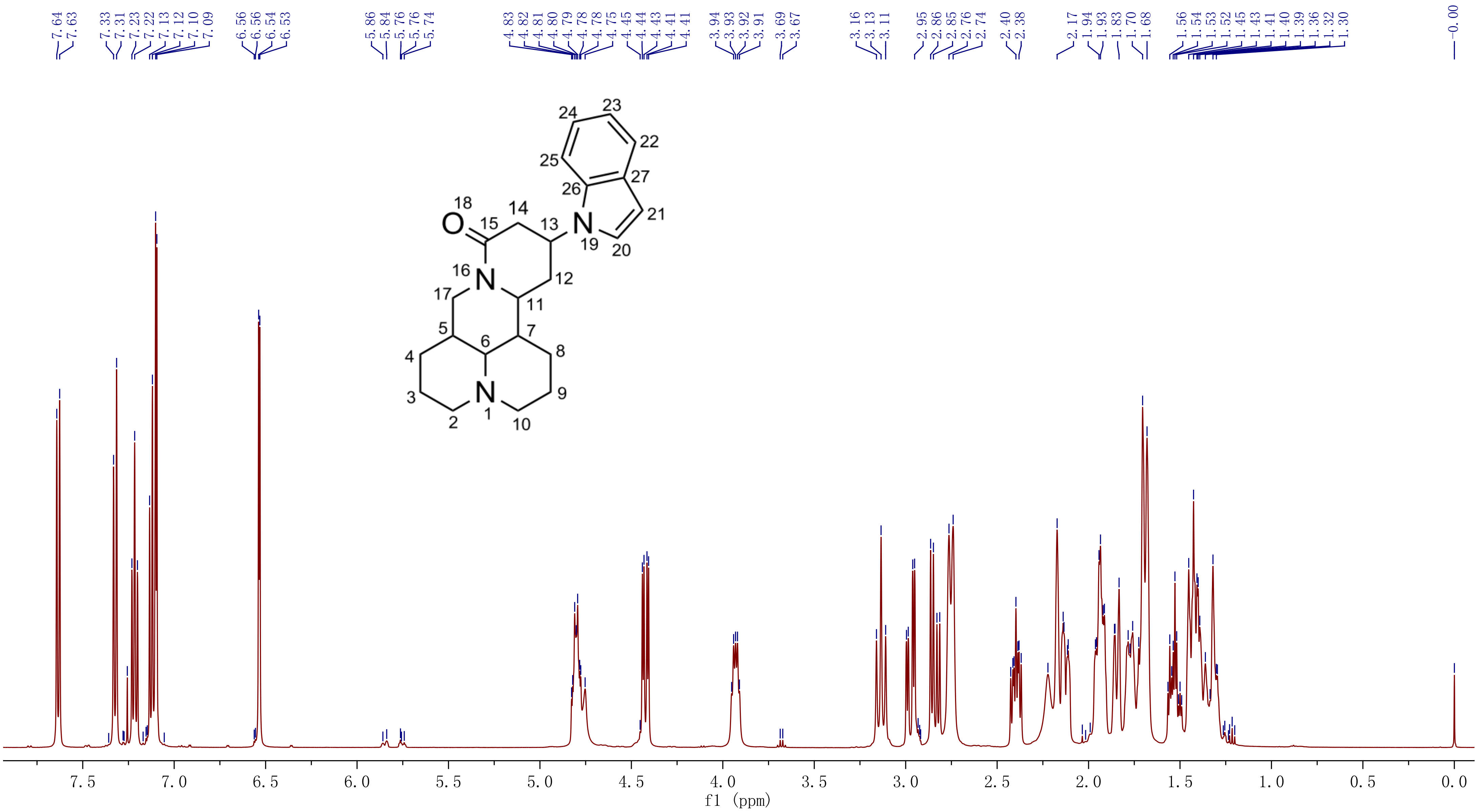

B

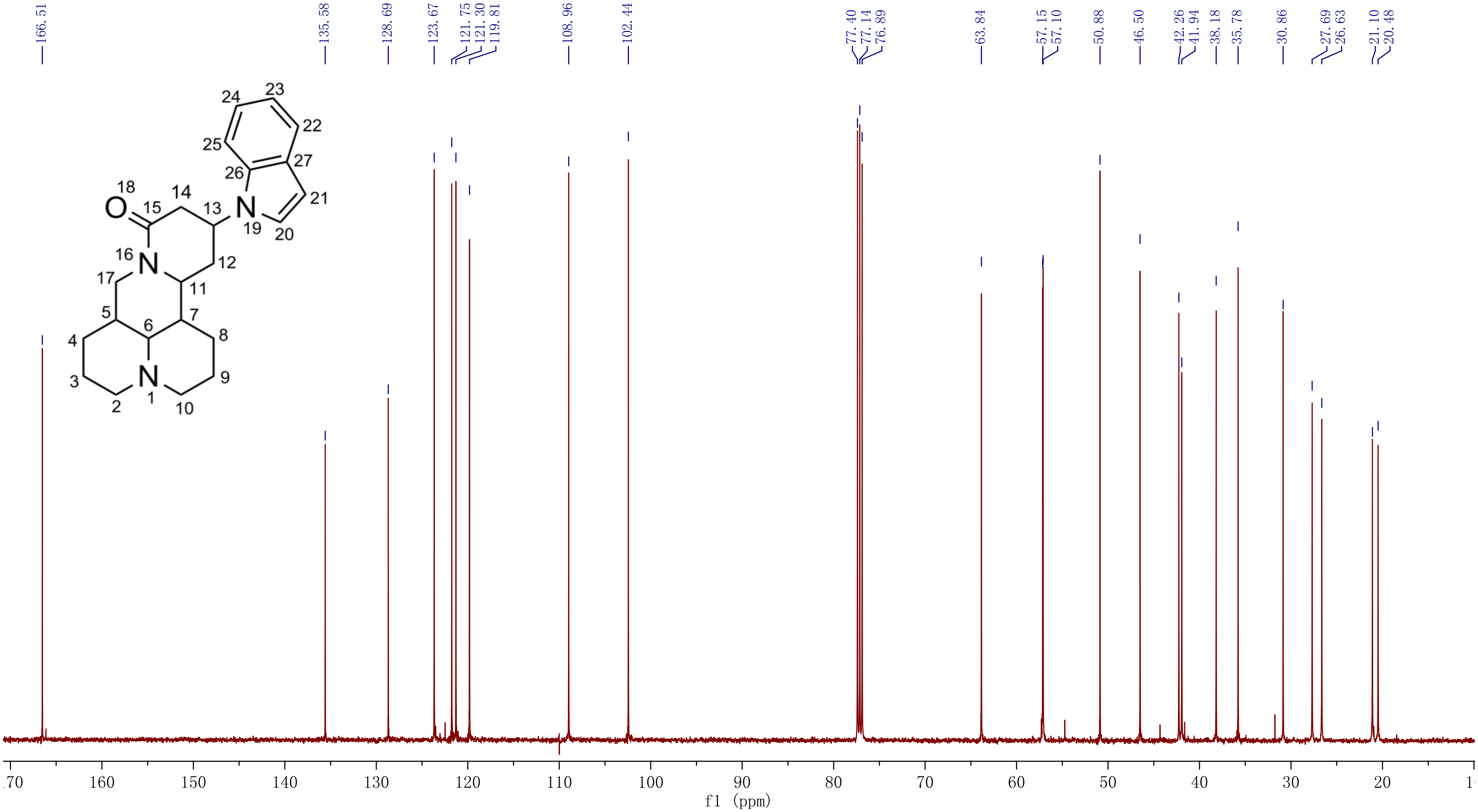
166.51
135.58
128.69
123.67
121.75
121.30
119.81
108.96
102.44
77.40
77.14
76.89
63.84
57.15
57.10
50.88
46.50
42.26
41.94
38.18
35.78
30.86
27.69
26.63
21.10
20.48
O
N
N
N
1 2 3 4 5 6 7 8 9 10 11 12 13 14 15 16 17 18 19 20 21 22 23 24 25 26 27
160 150 140 130 120 110 100 90 80 70 60 50 40 30 20
f1 (ppm)

Figure S3. ^{1}H NMR (A) and ^{13}C NMR (B) spectrums of matrine derivative 2 in $CDCl_3$

A

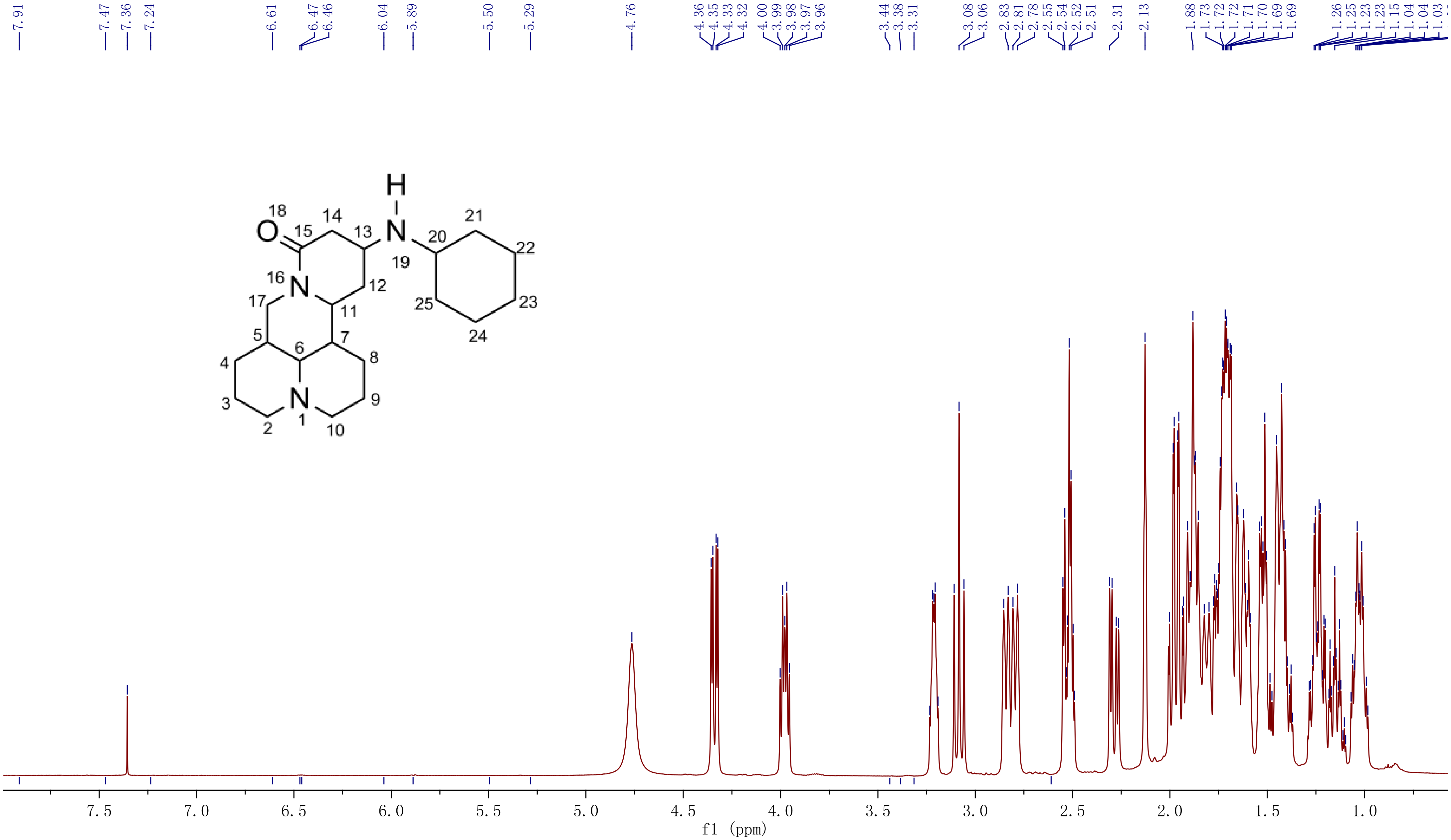

B

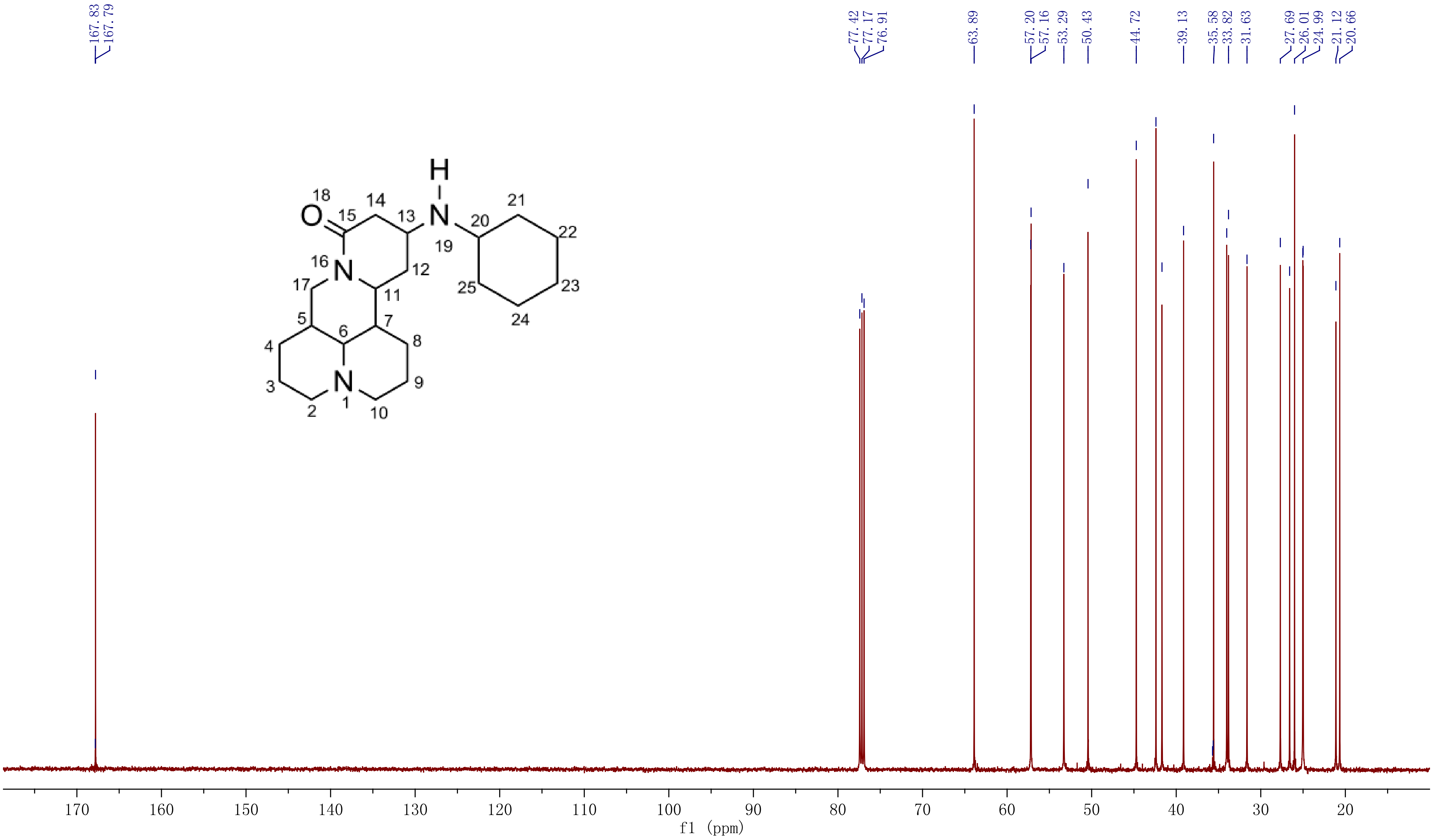
167.83
167.79
77.42
77.17
76.91
63.89
57.20
57.16
53.29
50.43
44.72
39.13
35.58
33.82
31.63
27.69
26.01
24.99
21.12
20.66
H
N
O
N
N
18
14
13
15
16
17
12
11
7
5
6
4
3
2
1
10
9
8
19
20
21
22
23
24
25
170
160
150
140
130
120
110
100
90
80
70
60
50
40
30
20
f1 (ppm)
